# A Nanograss Boron and Nitrogen Co-Doped Diamond Sensor Produced via High-Temperature Annealing for the Detection of Cadmium Ions

**DOI:** 10.3390/nano13222955

**Published:** 2023-11-15

**Authors:** Xiaoxi Yuan, Yaqi Liang, Mingchao Yang, Shaoheng Cheng, Nan Gao, Yongfu Zhu, Hongdong Li

**Affiliations:** 1 State Key Laboratory of Superhard Materials, College of Physics, Jilin University, Changchun 130012, China; xxyuan@jlenu.edu.cn (X.Y.); yqliang22@mails.jlu.edu.cn (Y.L.); chengshaoheng@jlu.edu.cn (S.C.); 2 Institute for Interdisciplinary Quantum Information Technology, Jilin Engineering Normal University, Changchun 130052, China; 3 Department of Physics, Hebei Normal University of Science and Technology, Qinhuangdao 066000, China; mcyang3968@hevttc.edu.cn; 4 Key Laboratory of Automobile Materials, Ministry of Education, School of Materials Science and Engineering, Jilin University, Changchun 130022, China

**Keywords:** diamond, nanograss, co-doping, electrochemical, detection, cadmium

## Abstract

The high-performance determination of heavy metal ions (Cd^2+^) in water sources is significant for the protection of public health and safety. We have developed a novel sensor of nanograss boron and nitrogen co-doped diamond (NGBND) to detect Cd^2+^ using a simple method without any masks or reactive ion etching. The NGBND electrode is constructed based on the co-doped diamond growth mode and the removal of the non-diamond carbon (NDC) from the NGBND/NDC composite. Both the enlarged surface area and enhanced electrochemical performance of the NGBND film are achievable. Scanning electron microscopy, Raman spectroscopy, X-ray photoelectron spectroscopy, cyclic voltammetry, electrochemical impedance spectroscopy, and differential pulse anodic stripping voltammetry (DPASV) were used to characterize the NGBND electrodes. Furthermore, we used a finite element numerical method to research the current density near the tip of NGBND. The NGBND sensor exhibits significant advantages for detecting trace Cd^2+^ via DPASV. A broad linear range of 1 to 100 μg L^−1^ with a low detection limit of 0.28 μg L^−1^ was achieved. The successful application of this Cd^2+^ sensor indicates considerable promise for the sensitive detection of heavy metal ions.

## 1. Introduction

Heavy metal contamination has become a severe global environmental problem with the development of agriculture and industry. Cd^2+^, as one of the most poisonous and prevalent heavy metal ions, is widely distributed and abundant in numerous environmental systems [1,2]. Some major sources of exposure to it are smoking and consumption of food, but inhalation of cadmium-containing dust is the most dangerous route [3]. Cadmium can be found in electroplated steel, pigments in plastics, in electric batteries, and so on [4]. Cd^2+^ is easily accumulated in the human body through the food chain due to its non-degradable properties [5]. Cd^2+^ exposure may result in serious cancer risks and organ injuries such as renal dysfunction, hypertension, teratogenic consequences, immune system damage, skeletal lesions, and so on [6]. Cd^2+^ accumulation can cause major health hazards, even at low levels in the human body, due to its extended half-life and lack of biodegradability. The maximum concentration of Cd^2+^ in drinking water is 3 μg L^−1^, according to the World Health Organization [7,8]. Therefore, it is essential to find efficient and sensitive methods of detecting Cd^2+^.

Many traditional analytical techniques for monitoring Cd^2+^ have been developed, such as inductively coupled plasma mass spectroscopy (ICP-MS), inductively coupled plasma-atomic emission spectrometry (ICP-AES), atomic fluorescence spectroscopy (AFS), atomic absorption spectroscopy (ASS), ion chromatography ultraviolet-visible spectroscopy (IC-UV-vis), X-ray fluorescence spectroscopy (XFS), and high-performance liquid chromatography (HPLC) [9,10,11,12]. These detection techniques can be performed with great resolution and accuracy, but their methods suffer from several limitations, including specialized equipment, complex workflows, and high cost. Thus, they are not suitable for the routine in-field monitoring of Cd^2+^ in real time. It is essential to develop a quick, easy, and accurate method for detecting Cd^2+^.

The electrochemical test is a powerful approach to detecting Cd^2+^ because of its accuracy, simplicity, low cost, remarkable sensitivity, effectiveness in multiplexed detection, and capacity for on-site detection [13]. As the key to electrochemical techniques, electrode materials play a critical role in the sensitivity of sensing devices. Nanomaterials are intriguing materials for electrode modification due to their enormous surface area and modification potential [14]. The improved performance stems from the nanoscale design of electrode surfaces, which results in greater catalytic activity, increased conductivity, an active large surface area, and quick electric kinetics [15].

Among modified nanomaterials, doped diamond materials (with their eminent chemical stability [16,17,18,19,20], high conductivity, wide electrochemical potential window [21], chemical inertness [22], biocompatibility [23], low-noise characteristic, and high corrosion resistance [24]) have been proven to be excellent sensors. Additionally, the electrocatalytic activity of doped diamond can be further strengthened through doping/co-doping heteroatoms such as boron, nitrogen, and sulfur to increase electrochemical performance. Generally, sensors with a large specific surface area provide more active sites for reactions, thereby improving their sensitivity in electroanalysis [25]. Therefore, nanodiamonds have extremely broad application prospects in electrochemical sensors [26,27]. Electrochemical sensors based on nanodiamonds have been employed in simpler applications (such as the determination of metals) and more sophisticated ones (including the use of biological molecules) [28].

A glassy carbon electrode modified with nanodiamond particles and carbon nanofiber and covered with a poly film was developed by Kaçar et al. to detect L-ascorbic acid [29]. Foord et al. modified a glassy carbon electrode with nanodiamonds to detect Bisphenol-A [30]. The modified electrode was prepared by drop-casting the dispersion of nanodiamonds in water. However, the possible biosafety problems of nanodiamonds are also of great concern. For example, during the process of detection, nanodiamonds might fall off and remain in drinking water or human blood, introducing a new form of pollution. Nanodiamonds in human cells may lead to oxidative damage, cytotoxicity, and other irreversible damage [31]. Compared with nanodiamonds, nanostructured diamonds also have excellent electrochemical activity, but it is not easy for them to fall off and lead to secondary pollution; this means they can ensure safety and long-term stability.

Several methods for constructing nanostructured diamond electrodes have been reported recently [32]. Conventional top-down approaches mainly refer to obtaining a three-dimensional nanostructure by etching the diamond surfaces with a mask or an external source (plasma etching [33], catalytic etching [34], thermal etching [35], etc.). However, the economic infeasibility of these preparation methods, such as complicated pretreatment or mask removal during the process, hinders subsequent extensive applications. Herein, we explore a nanograss boron-nitrogen-doped diamond (NGBND) electrode based on the investigation of doped diamonds’ growth mode and the ratio of diamond to non-diamond carbon (NDC).

In this paper, a simple and economical route for fabricating NGBND is demonstrated; this is achieved by removing NDC from the NGBND/NDC composite without any templates. We aim to use the NGBND electrode for electrochemically detecting Cd^2+^. The NGBND exhibits a large specific surface upon scanning electron microscopy (SEM). To better investigate the growth mechanisms of NGBND, the surface was characterized using Raman and X-ray photoelectron spectroscopy (XPS) measurements. Compared with electrochemical techniques, differential pulse anodic stripping voltammetry (DPASV) is an in situ electrochemical approach for the measurement of trace Cd^2+^ owing to its powerful advantages of high sensitivity, rapid analysis, and instrumental portability [36]. The NGBND exhibits great efficacy in the determination of trace Cd^2+^ using DPASV. Furthermore, we used a finite element numerical method to research the prospects of tip-enhanced current density. Overall, it presented significant advantages in the determination of trace Cd^2+^ based on an NGBND electrode. A wide linear range from 1 to 100 μg L^−1^ and a low detection limit of 0.28 μg L^−1^ were achieved in the detection of Cd^2+^, indicating its great potential for the sensitive detection of heavy metal ions.

## 2. Materials and Methods

### 2.1. Materials

The Cd(NO_3_)_2_ (99.9%) powders were guaranteed reagents obtained from Sigma-Aldrich (St. Louis, MO, USA). A 0.1 M acetate buffer was prepared with sodium acetate, and glacial acetic acid was used throughout the determination experiment. Other chemical reagents such as potassium ferricyanide (II), potassium ferrocyanide trihydrate (III), and potassium chloride were of analytical grade without further purification. Ultrapure water (18.2 MΩ·cm) was used to prepare aqueous solutions for all experiments.

### 2.2. Preparation of the NGBND Electrode

The diamond films were prepared on p-type Si substrates using a microwave plasma chemical vapor deposition system at 2.45 GHz. Before the deposition of diamond films, the mirror-polished substrates were ultrasonicated in an acetone solution with nano-diamond powders (about 5 nm) for 60 min to form nucleation sites. Then, the substrates were ultrasonic cleaned with acetone, ethanol, and purified water for 10 min, respectively, and dried with nitrogen. The reaction gas sources included methane (CH_4_) and hydrogen (H_2_). The liquid trimethyl borate (B(OCH_3_)_3_) was carried by bubbling H_2_ gas as the boron source. Firstly, the CH_4_/H_2_/B/N_2_ flow rate was set at 20/200/2/1 sccm to create a composite containing NGBND and NDC (NGBND/NDC composite) for 6 h. Secondly, the NGBND was fabricated to remove the NDC by annealing in a quartz tube at 800 °C for 20 min in the air.

### 2.3. Apparatus

SEM (JSM-6480LV, Akishima, Japan) was conducted to characterize the surface morphology of the NGBND films. The carbon phase composition was investigated via Raman spectroscopy (i.e., Renishaw in a Via Raman microscope, London, UK) using laser excitation at 532 nm. The surface bonds and the surface chemical states could be recorded using X-ray photoelectron spectroscopy (XPS, VG ESCALAB MK II, Dewsbury, Britain). All electrochemical characterization of the NGBND films was carried out on an electrochemical workstation (CHI 760E, Shanghai, China).

### 2.4. Electrochemical Measurements

In the three-electrode system, the platinum wire and saturated calomel electrode served as the counter and reference electrodes, respectively. The NGBND electrode served as the working electrode. The geometric area of the NGBND electrode was 0.10 cm^2^. The electrochemical impedance spectroscopy (EIS) texts were measured in a solution containing 5 mM Fe(CN)_6_^3−/4−^ and 0.1 M KCl. The electrochemical measurements carried out in an acetate buffer of the NGBND electrode were investigated using cyclic voltammetry (CV) and DPASV with an electrochemical workstation at room temperature.

### 2.5. COMSOL Multiphysics Simulations

The free electron density on the NGBND electrodes near the electrode was simulated using COMSOL Multiphysics 5.6. The density of free electrons on the electrode under a given potential value was obtained using the ‘Electric currents’ module. The formula of electric field E is E = −∇V, which comes from the negative gradient of potential V. The electric conductivity of the NGBND electrode was taken to be 2 × 10^4^ S m^−1^ [37]. The current density (ρ) was calculated using Gauss’s law of electric fields, and the formula was ρ = ε_r_ε_0_∇·E. ε_0_ and ε_r_ represent the dielectric function of the vacuum and the dielectric function of the material, respectively.

## 3. Results

### 3.1. Morphology and Structure of NGBND Films

**Figure 1:**
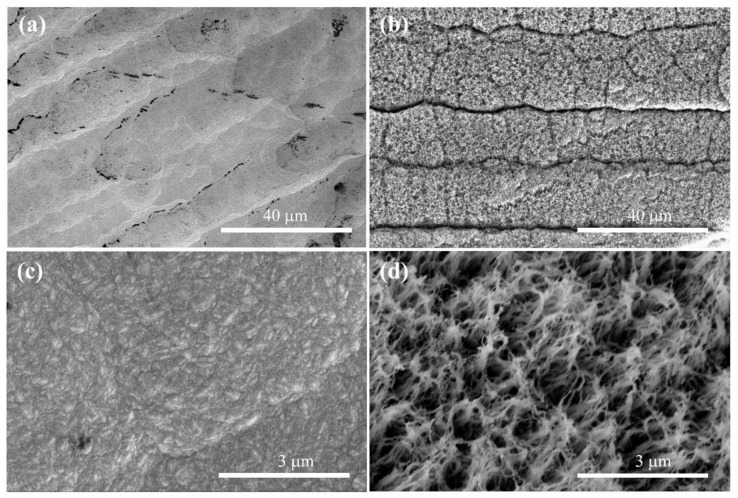
SEM images of the (**a**) NGBND/NDC composite film deposited with a CH_4_/H_2_/B/N_2_ flow rate of 20/200/2/1 sccm and the (**b**) NGBND film fabricated by etching the NDC phase (annealing in a quartz tube at 800 °C for 20 min in the air) from the composite. (**c**,**d**) are the images of (**a**,**b**), respectively, obtained at high magnification.

The black substance on the diamond surface might be NDC in Figure 1a. Due to the high proportion of CH_4_ introduced during the growth stage of the diamond, hydrogen plasma cannot completely etch off NDC. At the same time, nitrogen is introduced to facilitate the secondary nucleation of diamond, resulting in an unsmooth surface on the NGBND/NDC composite. Through etching the NDC phase from the NGBND/NDC composite, an NGBND film is obtained, and its SEM is presented in Figure 1b. The grain size of the NGBND/NDC composite is 200–300 nm (Figure 1c). As shown in Figure 1d, the nanograss is upward because of the columnar growth structure with the addition of nitrogen, and the tip size of NGBND is around a few nanometers. The morphology of NGBND is different from the nanoneedle boron-doped diamond without the addition of nitrogen (Figure S1 of the Supplementary Materials). The nanoneedle of BDD appears disorderly, which indicates that the addition of nitrogen plays a crucial role in the formation of nanograss diamonds. In comparison to previous methods of creating NGBND films, this method is more simple, low-cost, and efficient due to its controllable process, lack of complex template use, and the absence of expensive etching equipment.

**Figure 2:**
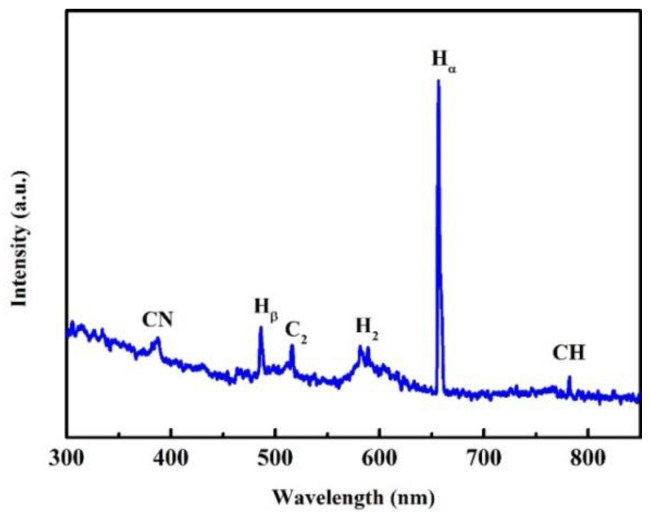
OES spectrum of the growth stage of the NGBND/NDC composite.

The OES results of the growth stage of the NGBND/NDC composite film are shown in Figure 2. The emission lines from the atomic hydrogen of H_α_ (656 nm) and H_β_ (486 nm), the molecular hydrogen of H_2_ (580 nm), the carbonaceous CN (386 nm) bands, the carbonaceous CH (432 and 766 nm) bands, and the C_2_ (516 nm) bands are presented. When the concentration of CH_4_ is high, a large amount of carbon carbonaceous C_2_ bands are generated, leading to the imperfect growth of diamonds with NDC. We used this mixed-growth method of producing diamonds and NDC to obtain the composite. The CN bands also form secondary nucleation during the growth of diamonds complemented with NDC. This is exactly why we need diamond grains that cannot grow upward. The NDC is then removed from the composite to obtain the NGBND. Moreover, abundant CN plays an important role in the columnar formation of nanograss.

**Figure 3:**
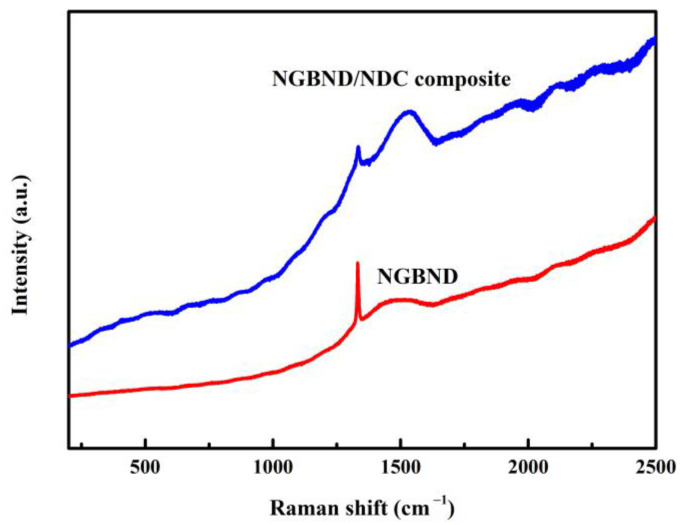
Raman spectra of the NGBND/NDC composite film and NGBND film.

The assumption that the quality of the NGBND/NDC composite film is poor is supported by the corresponding Raman spectrum (the blue line in Figure 3). A broad band centered at 1550 cm^−1^ is related to the NDC for a high concentration of CH_4_ during the process of diamond growth, and the characteristic diamond peak is weak at 1332 cm^−1^. After the annealing treatment, the NDC between the composite is etched away, and the NGBND is formed. The Raman spectrum of NGBND features a prominent diamond peak at 1332 cm^−1^ (red line in Figure 3). The broad band of NDC weakens after annealing. The residual broad peak might be attributed to some grain boundaries containing amorphous carbon components in NGBND.

**Figure 4:**
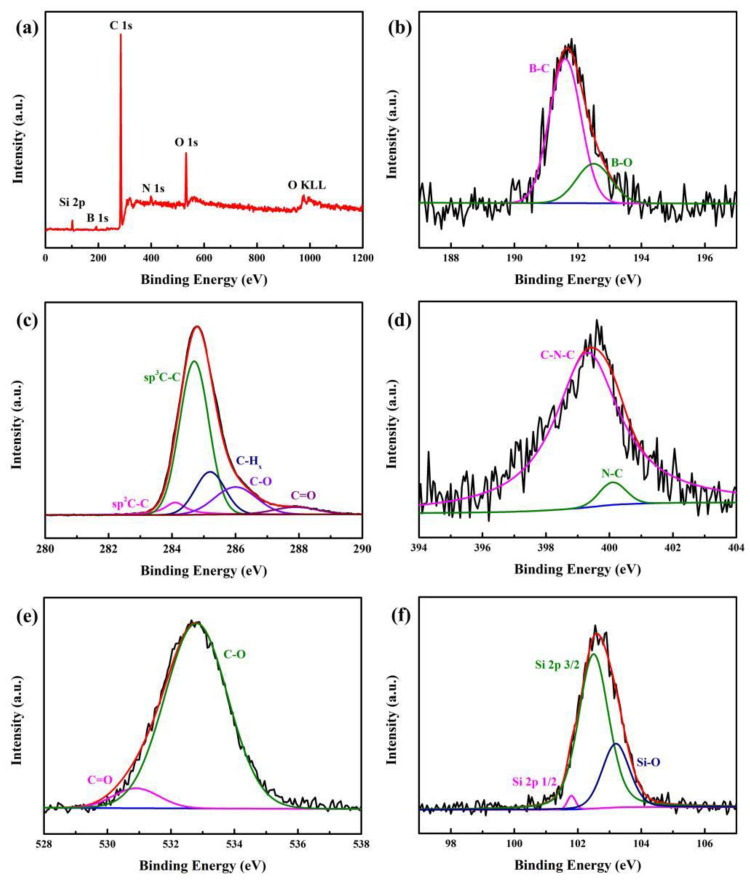
(**a**) Entire XPS scanning spectrum of NGBND. XPS high-resolution survey scan of (**b**) B 1s, (**c**) C 1s, (**d**) N 1s, (**e**) O 1s, and (**f**) Si 2p of NGBND. The black, red, and blue line is the experimental data, overall fit and background line in the subfigures (**b**–**f**).

For a better insight into NGBND, XPS measurements are shown in Figure 4. The peaks at 102.6 eV, 191.7 eV, 284.6 eV, 399.6 eV, 532.8 eV, and 975.0 eV represent the binding energies of Si 2p, B 1s, C 1s, N 1s, O 1s, and O KLL, respectively. This indicates that NGBND contains Si, B, C, N, and O elements (Figure 4a), and their contents are 1.71%, 1.41%, 92.42%, 0.59%, and 3.88%, respectively. The Si element sensitively identified comes from the Si substrate for preparing diamond films. XPS high-resolution survey scan spectra of B 1s, C 1s, N 1s, O 1s, and Si 2p are represented sequentially in Figure 4b–f. In the B 1s spectrum, the two peaks at 191.5 and 192.5 eV are ascribed to B–C and B–O bonds [38]. From the high-resolution of the C 1s spectrum, the peaks at 284.1, 284.5, 285.3, 286.3, and 287.8 eV are attributed to sp^2^ C–C, sp^3^ C–C, C–H_x_, C–O, and C=O, respectively [39,40,41]. Within the XPS analysis of the N 1s spectrum, two fitted peaks at 399.4 and 400.2 eV can be assigned to C–N–C and N–C [42]. Two oxygen species of the unsaturated C=O component at 530.9 eV and the saturated C-O component at 532.9 eV can be identified in the O 1s spectra [43]. After the annealing treatment, a large amount of carbon-oxygen bonds form due to the oxygen in the air, which bonds easily to the NGBND surface at high temperatures. Three synthetic peaks are applied to the Si 2p with the Si 2p3/2, Si 2p1/2, and Si–O at 101.8, 102.5, and 103.3 eV [43,44]. The Si–O bond originates from the formation of silicon oxide on the Si substrate during annealing at high temperatures. The absence of the Si-C bond indicates that the silicon element has not been doped in diamond, despite the Si element being detected within the XPS spectrum.

### 3.2. Electrochemical Performance

**Figure 5:**
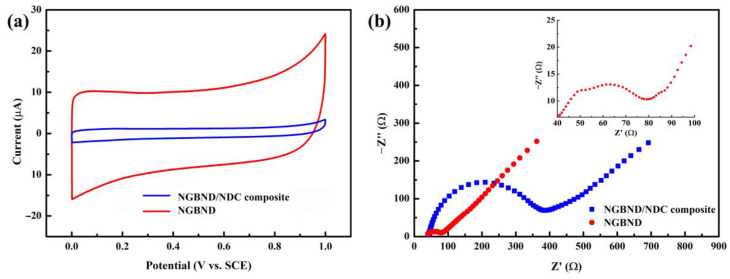
(**a**) CV curves of the NGBND/NDC composite and NGBND electrodes in 0.1 M acetate buffer at scan rates of 50 mV s^−1^. (**b**) EIS of NGBND/NDC composite and NGBND electrodes tested in a 5 mM Fe(CN)_6_^3−/4−^ solution containing 0.1 M KCl. The insert graph is a locally enlarged EIS image of NGBND.

The CV tests of the NGBND/NDC composite electrode and the NGBND electrode in Figure 5a prove that the estimated area of the NGBND electrode is 8.5 times that of the NGBND/NDC composite electrode, meaning that the NGBND structure provides more active sites for electrochemical detection. The EIS results in Figure 4b present the electron transfer kinetics of the NGBND/NDC composite electrode and the NGBND electrode. Compared with the NGBND/NDC composite electrode, the NGBND with the lower charge transfer resistance demonstrates that NGBND has a faster charge transfer rate at the interface between the electrode and solution. There are two semicircles in the EIS image insert in Figure 5b. The additional high-frequency semicircle of the NGBND electrode is related to the charge transfer resistance caused by the geometric morphology of the surface [45], which is consistent with the surface structure observed via SEM; meanwhile, the low-frequency semicircle is related to the charge transfer resistance of the Faraday reaction of the electrode. The large specific surface area and better charge transfer ability of NGBND may help to improve its effectiveness in electrochemical detection.

### 3.3. Electrochemical Characterization for Detecting Cd^2+^

**Figure 6:**
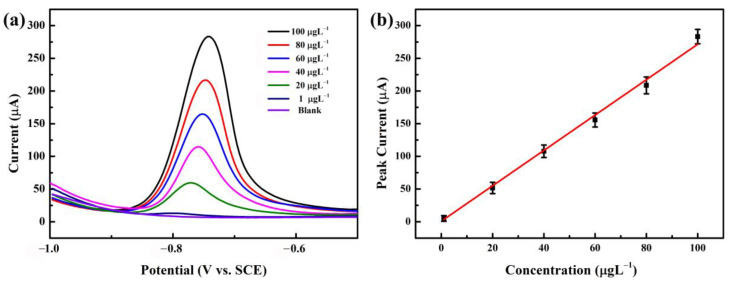
(**a**) DPASV diagrams of Cd^2+^ with concentrations between 1 and 100 μg L^−1^ on the NGBND electrode. (**b**) Calibration curve for Cd^2+^ detection. The error bars represent the relative standard deviations of triple measurements. The buffer used is 0.1 M acetate buffer (pH = 5.5).

Under pre-deposition accumulation conditions at a pH of 5.5, a deposition potential of −1.0 V, and a determination time of 270 s for Cd^2+^, a DPASV analysis was carried out on the NGBND electrode using a Cd^2+^ standard solution with different concentrations. Figure 6a shows a selection of typical DPASV curves for Cd^2+^ in the range of 1–100 μg L^−1^. Within the concentration range, the stripping peak shifted slightly to a more positive potential with increasing concentrations. For example, the peak potentials for 40, 60, 80, and 100 μg L^−1^ Cd^2+^ were −0.758, −0.751, −0.747, and −0.741 V, respectively. These results are consistent with those of previous studies showing that the phenomenon is due to the increasing equilibrium reduction potential of M^n+^/M and the enlarged size of metal particles deposited on the NGBND electrode surface with an increased ion concentration [46,47]. The stripping peaks obtained on the NGBND electrode were asymmetric and are attributed to the heterogeneously active electrode, in which the different graphically oriented grains are characterized by different electrical conductivity and surface structures.

The calibration plot in Figure 6b is linear, with good correlation coefficients. The sensitivity is within the range of 1 to 100 μg L^−1^ for Cd^2+^. Based on the response, which is three times the standard deviation of the zero-pose response, the limit of detection is 0.28 μg L^−1^. These results verify that the NGBND film may potentially be utilized for Cd^2+^ detection. Compared to the reported diamond electrodes used for detecting Cd^2+^ and listed in Table 1, the NGBND electrode has a good linear range and a greater detection limit than the other diamond electrodes [48,49,50,51,52,53,54,55].

### 3.4. Simulations of Current Density near the NGBND Tip

**Figure 7:**
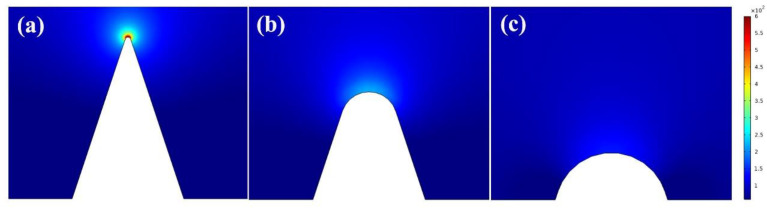
Current density distributions on the surface of NGBND at the electrode tip, which increase as the tip radius decreases. The tip radius of the structure in each panel is (**a**) 5 nm, (**b**) 50 nm, and (**c**) 100 nm.

Furthermore, to gain a deeper insight into the stripping process of Cd^2+^ deposits, we simulated the current density distribution within the vicinity of the NGBND electrode using COMSOL Multiphysics. To estimate the quantitative impact of the high-curvature structure on the current density, the current density directly adjacent to the electrode surface was mapped. Cones with rounded tips were used to represent the sharp tips of NGBND immersed in the electrolyte, and the current density distribution values around the electrode with a tip radius of 5, 50, or 100 nm are given in Figure 7. The maximum current density around the electrode tip is 6.67 × 10^2^, 2.21 × 10^2^, and 1.42 × 10^2^ A m^−2^, respectively. The maximum current density increases by 4.7 times as the tip radius of the electrode sharpens from 100 nm to 5 nm. This shows that the high-curvature structure can significantly enhance the current density, which might increase the Cd^2+^ concentration near the NGBND tip at low Cd^2+^ concentrations. The simulation results indicate that enhanced current density at high curvature sites may facilitate the precipitation of Cd^2+^ and the detection of Cd^2+^ at low concentrations via DPASV.

### 3.5. Reproducibility and Selectivity of NGBND

Reproducibility is an important indicator that reflects the precision of an electrode. Improving electrode reproducibility is critical to encouraging its use in online monitoring [56]. The relative standard deviation (RSD) value of the stripping peak current value from six repetitive experiments is used to evaluate the reproduction of the NGBND electrode after repeated detections of 100 μg L^−1^. A steady potential of +0.5 V is supplied to eliminate the residual from the NGBND electrode for 600 s following each measurement. The stripping peak current value of Cd^2+^ reduces slightly as the detection number increases without a shift in the stripping peak potential. The RSD value of the NGBND electrode is calculated to be 3.1%, indicating that NGBND has satisfactory reproducible precision.

Selectivity indicates the electrode’s anti-interference capacity for performance in complicated water environments [57]. Several interfering ions, including Pb^2+^, Zn^2+^, Ca^2+^, Cu^2+^, Mg^2+^, and Na^+^, were individually added to a standard solution of Cd^2+^ with a concentration ten times that of Cd^2+^. As demonstrated in Figure S2, the signal of Cd^2+^ changed slightly when the ions of Pb^2+^, Zn^2+^, Ca^2+^, Cu^2+^, Mg^2+^, and Na^+^ were added. Tests of the individual metal ions’ sensor performance (Pb^2+^, Zn^2+^, Cu^2+^, Ca^2+^, Mg^2+^, and Na^+^) without mixing Cd^2+^ are presented in Figure S3. This indicates that the NGBND electrode has better anti-interference properties for the six ions above. Recycling tests of the sensor were measured seven times in the Cd^2+^ solution. There are variations in the peak current responses, which are localized in the region of −3.7–4.2%, compared with the first test.

To realize the possible practical applications of Cd^2+^ detection, the NGBND sensor was applied to water samples collected from different lakes in Changchun, China. The samples were filtered through a membrane. Water without Cd^2+^ was selected as the control group. The results of the water samples with different concentrations of Cd^2+^ are summarized in Table 2. The sensor was proven to be able to detect trace amounts of Cd^2+^ with recoveries of 97–104% and relative standard deviations less than 3.8%.

## 4. Conclusions

In summary, NGBND films were synthesized in an H_2_/CH_4_/B/N_2_ source gas mixture and annealed in the air using a simple method without any masks or reactive ion etching. An NGBND electrode with an enlarged surface area was constructed based on a co-doped diamond growth mode and the removal of NDC from an NGBND/NDC composite. The NGBND electrode sensor exhibits good linearity from 1–100 μg L^−1^ and a good detection limit of 0.28 μg L^−1^ for the determination of Cd^2+^. It was demonstrated that an enlarged specific surface area allows for more electrochemically active points, and the enhanced current density of the sharp tip may work to increase the local Cd^2+^ concentration near the NGBND electrode, thereby playing an important role in accomplishing an excellent detection limit. The NGBND sensor shows high reproducibility and selectivity for detecting trace Cd^2+^. This sensor could potentially be utilized for sensitively detecting other substances such as heavy metal ions, biomolecules, drugs, environmental hazards, pesticides, organic molecules, etc.

## Figures and Tables

**Table 1:** Relevant diamond electrodes for the detection of Cd^2+^.

Electrodes	Technique	Linear Range (μg L^−1^)	Detection Limit (μg L^−1^)	Relative Standard Deviation	Ref.
BDD ^1^	SWASV ^3^	2–40	1.6	—	[48]
hydrogen-terminated BDD	SWASV	5.6–448	3.38	1.6%	[49]
Sb/BDD	LSASV ^4^	100–500	38.1	—	[50]
NDD ^2^	SWASV	1.1–123.2	1.1	—	[51]
Bi/BDD	SWASV	20–200	2.3	—	[52]
Bi/nitrogen carbon/BDD	DPASV ^5^	1–10	0.51	3.4%	[53]
graphite/diamond	DPASV	5–1000	0.47	14.4%	[54]
diamond/carbon nanowalls	DPASV	9.97–996.8	9.97	—	[55]
NGBND	DPASV	1–100	0.28	3.1%	This work

^1^ BDD: boron-doped diamond; ^2^ NDD: nitrogen-doped diamond; ^3^ SWASV: square-wave anodic stripping voltammetry; ^4^ LSASV: linear sweep anodic stripping voltammetry; ^5^ DPASV: differential pulse anodic stripping voltammetry.

**Table 2:** Recoveries of Cd^2+^ with varying concentrations added to lake water samples.

Lake Water	Added Cd^2+^ (μg L^−1^)	Detected (μg L^−1^)	Recovery (%)	Relative Standard Deviation (%)
1	20	19.6	97	3.8
2	40	41.5	104	3.2
3	60	61.1	102	2.6

## Data Availability

Data are contained within the article and supplementary materials.

## Supplementary Materials

The following supporting information can be downloaded at: https://www.mdpi.com/article/10.3390/nano13222955/s1, Figure S1: SEM image of nano needle boron-doped diamond; Figure S2: Bar graph of selective ability to resist ion interference; Figure S3: DPASV tests for individual metal ions without mixing Cd^2+^.
