# Supplementary material for: A Nanograss Boron and Nitrogen Co-Doped Diamond Sensor Produced via High-Temperature Annealing for the Detection of Cadmium Ions
Source: Nanomaterials (Basel). 2023 Nov 15;13(22):2955. doi: 10.3390/nano13222955 (PMC10675383; doi:10.3390/nano13222955)
Supplement: Supplementary file 1 [file nanomaterials-13-02955-s001.zip › nanomaterials-2689689-supplementary.pdf]

Supplementary Materials

A Nanograss Boron and Nitrogen Co-Doped Diamond Sensor Produced via High-Temperature Annealing for the Detection of Cadmium Ions

Xiaoxi Yuan [1,2], Yaqi Liang [1], Mingchao Yang [3], Shaoheng Cheng [1], Nan Gao [1,*], Yongfu Zhu [4,*] and Hongdong Li [1,*]

[1] State Key Laboratory of Superhard Materials, College of Physics, Jilin University, Changchun 130012, China; xxyuan@jlenu.edu.cn (X.Y.); yqliang22@mails.jlu.edu.cn (Y.L.); chengshaoheng@jlu.edu.cn (S.C.)

[2] Institute for Interdisciplinary Quantum Information Technology, Jilin Engineering Normal University, Changchun 130052, China

[3] Department of Physics, Hebei Normal University of Science and Technology, Qinhuangdao 066000, China; mcyang3968@hevttc.edu.cn

[4] Key Laboratory of Automobile Materials, Ministry of Education, School of Materials Science and Engineering, Jilin University, Changchun 130022, China

* Correspondence: gaon@jlu.edu.cn (N.G.); yfzhu@jlu.edu.cn (Y.Z.); hdli@jlu.edu.cn (H.L.)

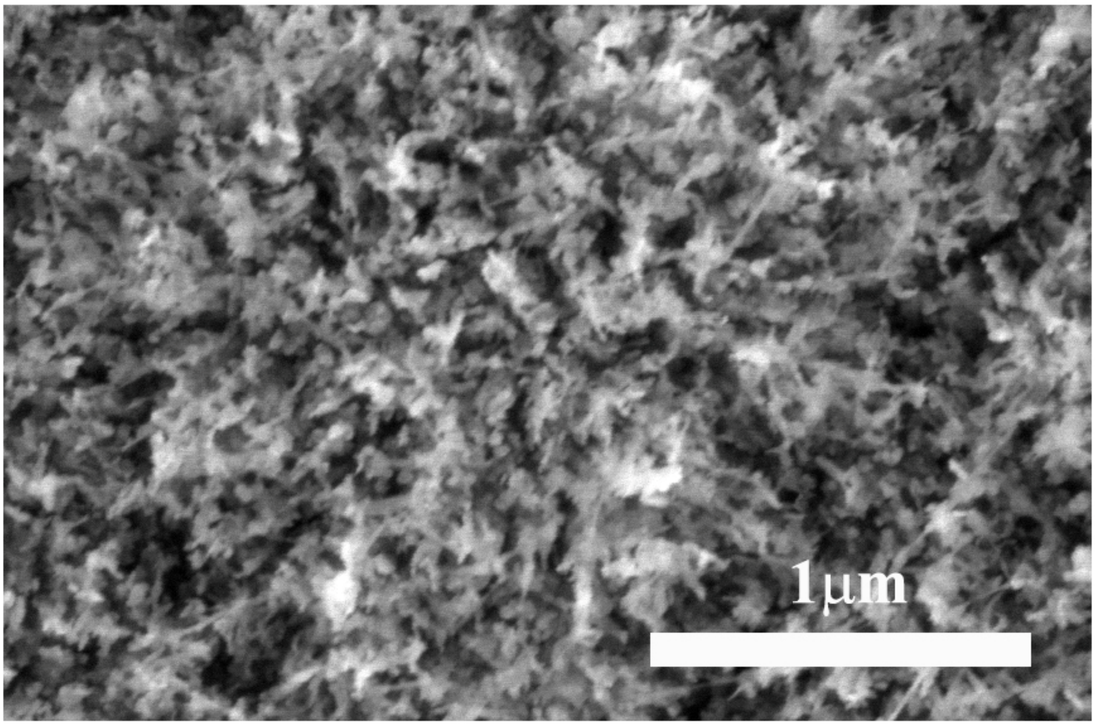

Figure S1. SEM images of boron doped diamond with nano needle.

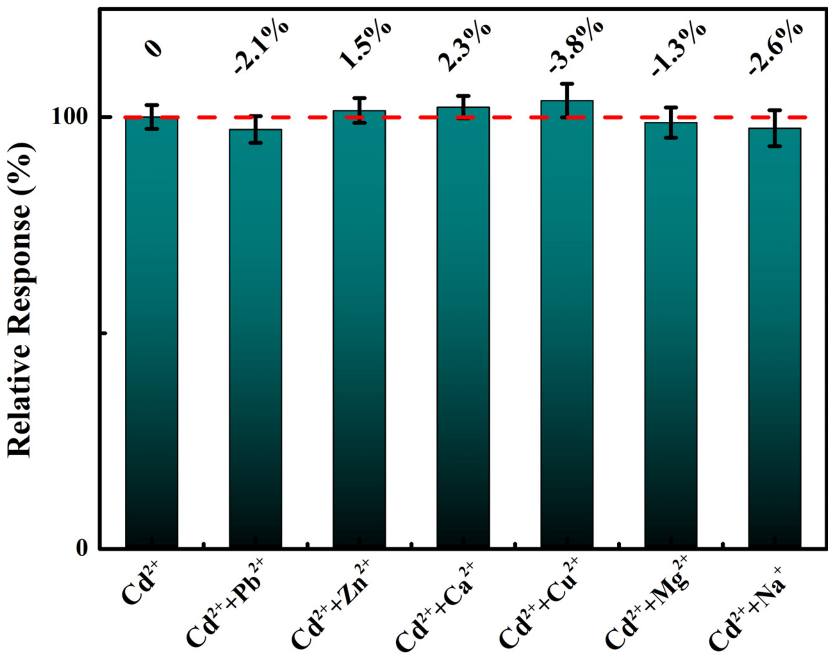

Figure S2. Bar graph of selective ability to resist ion interference.

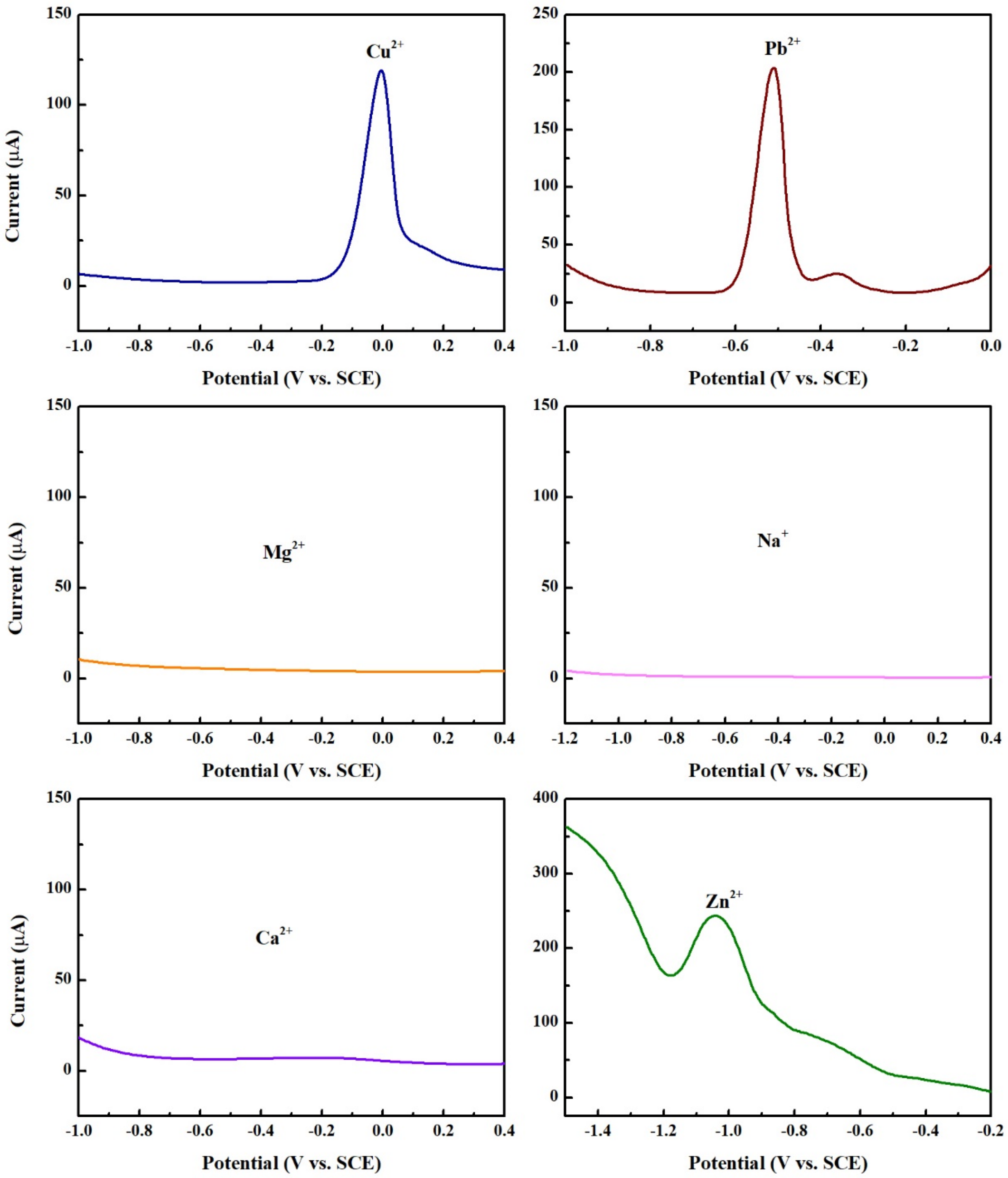

Figure S3. DPASV tests for individual metal ions without mixing Cd^{2+}.
